# Autophagy in the Vertebrate Inner Ear

**DOI:** 10.3389/fcell.2017.00056

**Published:** 2017-05-26

**Authors:** Marta Magariños, Sara Pulido, María R. Aburto, Rocío de Iriarte Rodríguez, Isabel Varela-Nieto

**Affiliations:** ^1^Department of Endocrine and Nervous Systems Pathophysiology, Instituto de Investigaciones Biomédicas “Alberto Sols,” CSIC-UAMMadrid, Spain; ^2^CIBERER, Unit 761, Instituto de Salud Carlos IIIMadrid, Spain; ^3^Departamento de Biología, Universidad Autónoma de MadridMadrid, Spain; ^4^Instituto de Investigación Hospital Universitario La Paz (IdiPAZ)Madrid, Spain

**Keywords:** Atg4, Atg5, Beclin-1, cochlea, LC3, otic development, vestibular system

## Abstract

Autophagy is a conserved catabolic process that results in the lysosomal degradation of cell components. During development, autophagy is associated with tissue and organ remodeling, and under physiological conditions it is tightly regulated as it plays a housekeeping role in removing misfolded proteins and damaged organelles. The vertebrate inner ear is a complex sensory organ responsible for the perception of sound and for balance. Cell survival, death and proliferation, as well as cell fate specification and differentiation, are processes that are strictly coordinated during the development of the inner ear in order to generate the more than a dozen specialized cell types that constitute this structure. Here, we review the existing evidence that implicates autophagy in the generation of the vertebrate inner ear. At early stages of chicken otic development, inhibiting autophagy impairs neurogenesis and causes aberrant otocyst morphogenesis. Autophagy provides energy for the clearing of dying cells and it favors neuronal differentiation. Moreover, autophagy is required for proper vestibular development in the mouse inner ear. The autophagy-related genes *Becn1, Atg4g, Atg5*, and *Atg9*, are expressed in the inner ear from late developmental stages to adulthood, and *Atg4b* mutants show impaired vestibular behavior associated to defects in otoconial biogenesis that are also common to *Atg5* mutants. Autophagic flux appears to be age-regulated, augmenting from perinatal stages to young adulthood in mice. This up-regulation is concomitant with the functional maturation of the hearing receptor. Hence, autophagy can be considered an intracellular pathway fundamental for in vertebrate inner ear development and maturation.

## An introduction to autophagy

Autophagy is a catabolic process that degrades the cytoplasmic content of a cell in lysosomes, returning energy, and molecular building bricks to the cell. Indeed, autophagy has a housekeeping role in cells as it is a way to eliminate damaged macromolecules, organelles, and pathogens. Since the initial description of autophagy by Christian de Duve in 1963, it has become more and more relevant as it has become implicated in a variety of physiological and pathological situations (Jiang and Mizushima, 2014). Indeed, three different types of autophagy are now recognized: (1) Macroautophagy (herein autophagy), where a double-membrane autophagosome forms and engulfs cytoplasmic content, subsequently fusing with the lysosome to form an autolysosome and releasing the autophagosome cargo into the lysosome lumen to be degraded by hydrolases; (2) Microautophagy, in which the cargo reaches the lumen by invagination of the lysosomal membrane; and (3) Chaperone-mediated autophagy, exclusive to mammals, where proteins associated to chaperones bind to the LAMP2A lysosomal receptor and are delivered directly to the lumen (Tasset and Cuervo, 2016).

**Figure 1:**
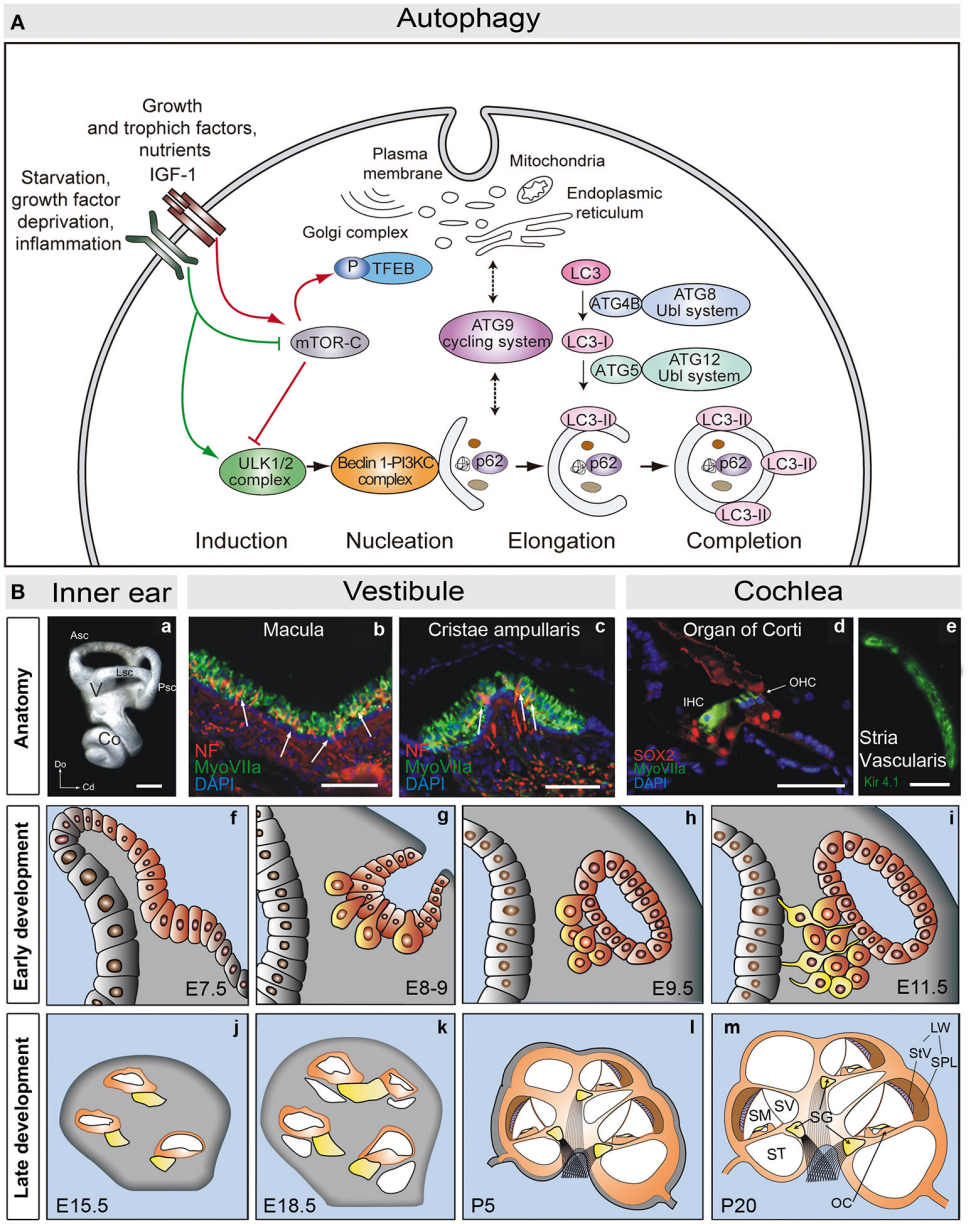
**(A)** Schematic view of the molecular steps of macroautophagy. Growth factors and nutrient-rich conditions activate mTORC1, a negative regulator of the ULK1/2 complex and TEFB. In turn, growth factor deprivation, inflammation, or nutrient starvation, activate the ULK1/2 complex, which phosphorylates and activates the PI3K complex III (PI3KC). The ATG9 cycling system provides membranes to form the autophagosome from different donor sources. Autophagosome formation also requires the action of two ubiquitin-like (Ubl) systems, ATG8-Ubl and ATG12-Ubl, required for the elongation and completion of the autophagosome. LC3 is converted into the cytosolic form, LC3-I, by cleavage of ATG4B, and into the membrane associated form, LC3-II, by conjugation with phosphoethanolamine via ATG5 (and the remaining components of the ATG12-Ubl system). SQSTM1/p62 (p62) binds to ubiquitinated proteins and carries them to the autophagosome (adapted from de Iriarte Rodríguez et al., 2015). **(B)** Anatomy of the adult mouse inner ear. **(a)** Lateral view showing a mammalian inner ear. **(b,c)** Detail of the vestibular macula **(b)** and cristae ampullaris **(c)**, where sensory hair cells are labeled for myosin VIIa (green) and neurofilament (red). **(d)** Detail of the organ of Corti showing myosin VIIa positive hair cells (green) and SOX2 positive supporting cells (red). **(e)** The stria vascularis is visualized by labeling for Kir4.1 (green). Development of the mouse inner ear. The inner ear develops from the otic placode (**f**, E7.5). The otic placode invaginates to form the otic cup (**g**, E8-9), which later pinches off to form the otic vesicle or otocyst **(h,i)**. Neural precursors delaminate from the ventral otocyst epithelium to form the acoustic-vestibular ganglion (AVG: **g–i**). The cochlear duct evaginates from the ventromedial region of the otic vesicle, and it will be innervated by the acoustic portion of the AVG, also known as the spiral ganglion (SG: yellow, **j–m**). The cochlear duct elongates and grows to form a coiled tube, the membranous labyrinth, which includes the primordium of the scalas media, vestibularis, and tympanic **(j–m)**. At the cochlear duct the prosensory patch will become the primitive organ of Corti. Scale bars: **(a)** 0.5 mm; **(b–e)** 50 μm. Co, cochlea; V, vestibule; Asc, Lsc and Psc, anterior, lateral and posterior semicircular canals; Do, dorsal; Cd, caudal; IHC, inner hair cells; OHC, outer hair cells; StV, stria vasculari; SpL, spiral ligament; SV, scala vestibule; SM, scala media; ST, scala tympani; LW, lateral wall; OC, Organ of Corti (adapted from Magariños et al., 2014).

The formation of the autophagosome requires the activity of a set of proteins, most of them encoded by the autophagy related genes (*ATG*; Figure 1A). The formation of the autophagosome involves induction, nucleation, elongation, and completion. A specific subset of ATG proteins has been associated to each of these stages (Ariosa and Klionsky, 2016). As such, the ULK1/2 complex (ATG13, ATG101, FIP200) participates in induction and ULK1 activates the phosphatidylinositol 3-kinase complex (PI3KC: Beclin-1, Vsp34, Vps15, ATG14) to promote nucleation. Two ubiquitin-like conjugation systems contribute to elongate the phagophore: ATG12 (ATG12, ATG7, ATG10, ATG5, and ATG16L) and ATG8 (LC3, the mammalian homolog of ATG8). Both these complexes regulate the formation of LC3-II, the relative levels of which serve as a readout of the autophagic flux, along with SQSTM1/p62 that facilitates the entry of the cargo into the autophagosome. Accordingly, the SQSTM1/p62 levels are inversely correlated with those of LC3-II (Katsuragi et al., 2015; Klionsky et al., 2016). Finally, the ATG9 cycling system incorporates membranes from cell donor locations (Pavel and Rubinsztein, 2016). Following the completion of the autophagosome, its fusion with lysosomes requires the activity of proteins involved in other vesicular fusion events, such as the SNARE (soluble NSF attachment protein receptor) and HOPS (homotypic fusion and vacuole sorting proteins) complexes (Zhen and Li, 2015).

Autophagy can be induced by starvation, growth factor deprivation, hypoxia, or infections. These stimuli elicit an immediate response and long-term gene expression responses mediated by specific transcription factors like TFEB (transcription factor EB). TFEB acts as a master regulator of autophagy by up-regulating the expression of autophagy genes. Under nutrient-rich conditions, TFEB is phosphorylated by mTORC1 (mammalian target of rapamycin complex (1) and kept inactivate in the cytosol, mTORC1 also inhibiting autophagy by phosphorylation of ATG13, Füllgrabe et al., 2016; Napolitano and Ballabio, 2016).

Autophagy is a housekeeping mechanism that removes damaged molecules and organelles from the cell's cytoplasm, yet it also participates in the immune response, and it provides energy and molecules as building blocks when needed. Autophagy is essential during development, as it contributes to organ and tissue sculpting in *Drosophila* by facilitating cell death (Denton et al., 2012). Indeed, autophagy may promote largescale cytosolic self-digestion and the removal of certain pro-survival proteins (Yu et al., 2006). Thus, the final output of autophagy could be either positive or negative for the cell, and this depends on the intensity and duration of its induction.

## Developmental autophagy

Autophagy contributes to developmental tissue remodeling, responding to specific extrinsic, and intrinsic stimuli. For example, following fertilization of the mouse egg, autophagy removes maternal mRNA and proteins, allowing the egg to initiate its zygotic program (Tsukamoto et al., 2008; Yamamoto et al., 2014). Later on in development, autophagy drives the development of the nervous system, adipose tissue, osseous tissue, hematopoietic system, and the heart (Aburto et al., 2012a). The study of genetically modified mice has shed light on the roles played by the genes involved in autophagy. *Ambra1* is an essential gene for the development of the mouse central nervous system, the deficiency of which impairs autophagy and induces aberrant neuronal proliferation (Fimia et al., 2007; Antonioli et al., 2015). Different mutations in genes that participate in the autophagy machinery have shown that autophagy is needed for terminal neuronal differentiation, and specifically for axonal outgrowth and guidance. For example, axon formation is disturbed in the cerebellar granule neurons of *Ulk1*^−/−^ mice (Zhou et al., 2007) and more recently, ALFY, an adaptor protein between the cargo and the ATG proteins, was seen to be required for axon outgrowth in the brain and to establish neuronal connectivity (Dragich et al., 2016).

*Atg* mutants have provided evidence that autophagy is needed for the correct development of adipose, osseous and cardiac tissues, as well as for the differentiation of hematopoietic cells. *Atg5 and Atg7* deficiency is associated with a reduction in thymocytes and B-lymphocytes (Pua et al., 2007), as well as reduced levels of adipocyte differentiation factors and decreased lipid adipose mass (Singh et al., 2009). In addition, *Atg5* and *Atg7* deficiency in the embryonic P19CL6 cells inhibited cardiac cell differentiation (Jia et al., 2014). However, in the analysis of the phenotypes associated to these mutations it should be considered that ATG proteins also fulfill functions that are not related to autophagy (Mauthe and Reggiori, 2016).

During development, autophagy facilitates rapid changes in intracellular composition, promoting the turnover of specific proteins, receptors, cytoskeletal components, or transcription factors necessary to define the different cell fates. It is also essential for the temporal dynamics of cell organelles, controlling their number, and quality (e.g., mitochondria). Finally, after birth and before the initiation of suckling behavior, the up-regulation of autophagy protects newborns from death by starvation (Kuma et al., 2004). Autophagy may not only supply energy at this stage but it may also help control oxidative stress (Schiaffino et al., 2008).

## An introduction to inner ear anatomy

The mammalian inner ear is a complex sensory structure within the temporal bone that is composed of the cochlea and the vestibule, structures that are responsible for the senses of hearing and balance, respectively (Figure 1B). The auditory and vestibular organs contain the mechanosensory receptors that transduce mechanical stimuli into electrochemical signals that are transmitted to the brain by the fibers of the VIIIth cranial nerve. The auditory receptor is the organ of Corti in the scala media of the cochlea (Magariños et al., 2012, 2014), which is formed by sensory hair cells and by non-sensory support cells (Deiters', Hensen's and Claudius') that maintain the ionic and metabolic cochlear homeostasis (Forge and Wright, 2002). There are two functional types of hair cells arranged in a stereotypic manner: one row of inner hair cells (IHC) and three rows of outer hair cells (OHC; Forge and Wright, 2002; Magariños et al., 2012). The IHC cells connect to bipolar auditory type I neurons of the spiral ganglion, whilst the OHC are innervated by type II neurons (Nayagam et al., 2011; Fritzsch et al., 2015). The axons of these neurons leave the spiral ganglion to form the cochlear division of the acoustic-vestibular nerve, which is responsible for transmitting the auditory information through a multisynaptic, ascendant pathway from the cochlea to the auditory cortex (Demanez and Demanez, 2003). HC stereocilia are bathed by endolymph, which maintains the unique ionic concentration required for mechanotransduction. The stria vascularis is located in the lateral wall of the scala media. This three-layered structure regulates cochlear ion transport and maintains the endocochlear potential (Patuzzi, 2011).

The vestibular system is formed by five sensory structures, three cristae located at the base of the semicircular canals and two maculae. Each of these structures has a similar organization, with sensory HC and non-sensory support cells innervated by the vestibular ganglion axons. The vestibule is responsible for sensing equilibrium, and for the perception of linear and angular acceleration, and of gravity (Highstein and Fay, 2004; Ekdale, 2016).

## The regulation of inner ear development by extracellular factors and intracellular signaling networks

The development of the inner ear is initiated by the induction of the otic placode from the ectoderm lying between the rhombomeres 5 and 6 (Magariños et al., 2014; Whitfield, 2015). Otic placode induction is orchestrated from mesoderm signals that coordinate with intrinsic factors in the ectoderm. FGFs, Notch and WNT signaling play a key role during these initial events (Ohyama et al., 2006, 2007; Jayasena et al., 2008). The otic placode then invaginates to form the otic cup that will later detach and close to form the otocyst or otic vesicle. The otocyst is transient embryonic round structure whose multipotent cells will differentiate to produce most adult inner ear cell types (Bissonnette and Fekete, 1996; Sanchez-Calderon et al., 2007). The ventral region of the otocyst is specified by the Sonic hedgehog (Shh) secreted from the floor plate and notochord (Riccomagno et al., 2002, 2005), as well as through repression by the WNT signaling pathway (Groves and Fekete, 2012). Significantly, it is this region that will form the auditory portion of the inner ear. The vestibule develops from the dorsal otocyst, instructed by signals from the bone morphogenetic protein BMP4 (Chang et al., 2008) that antagonize Shh. Sensory HC, non-sensory support cells, plus the acoustic and vestibular neurons that contribute to the acoustic-vestibular ganglion (AVG) also arise from the otocyst. Finally, Notch signaling helps specify the prosensory domain (Daudet and Lewis, 2005; Hartman et al., 2010) and in combination with *Atoh1* expression, it is involved in determining the HC and supporting cells (Mizutari et al., 2013).

Otic vesicle development requires the coordinated response to apoptosis, survival and proliferation signals. IGF-1 signaling, mainly through the RAF-MEK-ERK and PI3K/AKT pathways, fulfills a critical role in regulating these processes. In the chicken embryo, PI3K/AKT signaling regulates the number of otic neurons and it determines the timing of their generation (Aburto et al., 2012b). Moreover, both the RAF-MEK-ERK and PI3K/AKT pathways modulate AVG neuritogenesis (Magariños et al., 2010; Aburto et al., 2012b). Phosphatase and tensin homolog deleted on chromosome 10 (PTEN) is required to define the size of the neuroblast population (Kim et al., 2013) and it negatively regulates the AKT signaling pathway, as well as interacting with the WNT, Notch, and BMP pathways.

Cell cycle regulation is also essential for correct inner ear organogenesis. IGF-1, Notch, and WNT are among the signaling pathways involved in regulating the proliferation of otic progenitors (Magariños et al., 2014). Through the RAF-MEK-ERK pathway, IGF-1 promotes the cell cycle progression of otic progenitors (Sanz et al., 1999b; Magariños et al., 2010). Finally, the otocyst must undergo the morphogenetic changes that transform the simple pseudostratified otic vesicle epithelia into an extremely complex three-dimensional membranous labyrinth (Kelly and Chen, 2009). The neighboring mesenchymal cells will be responsible for generating the bony labyrinth (Chang et al., 2002).

## Developmental autophagy in the embryonic chicken inner ear

**Figure 2:**
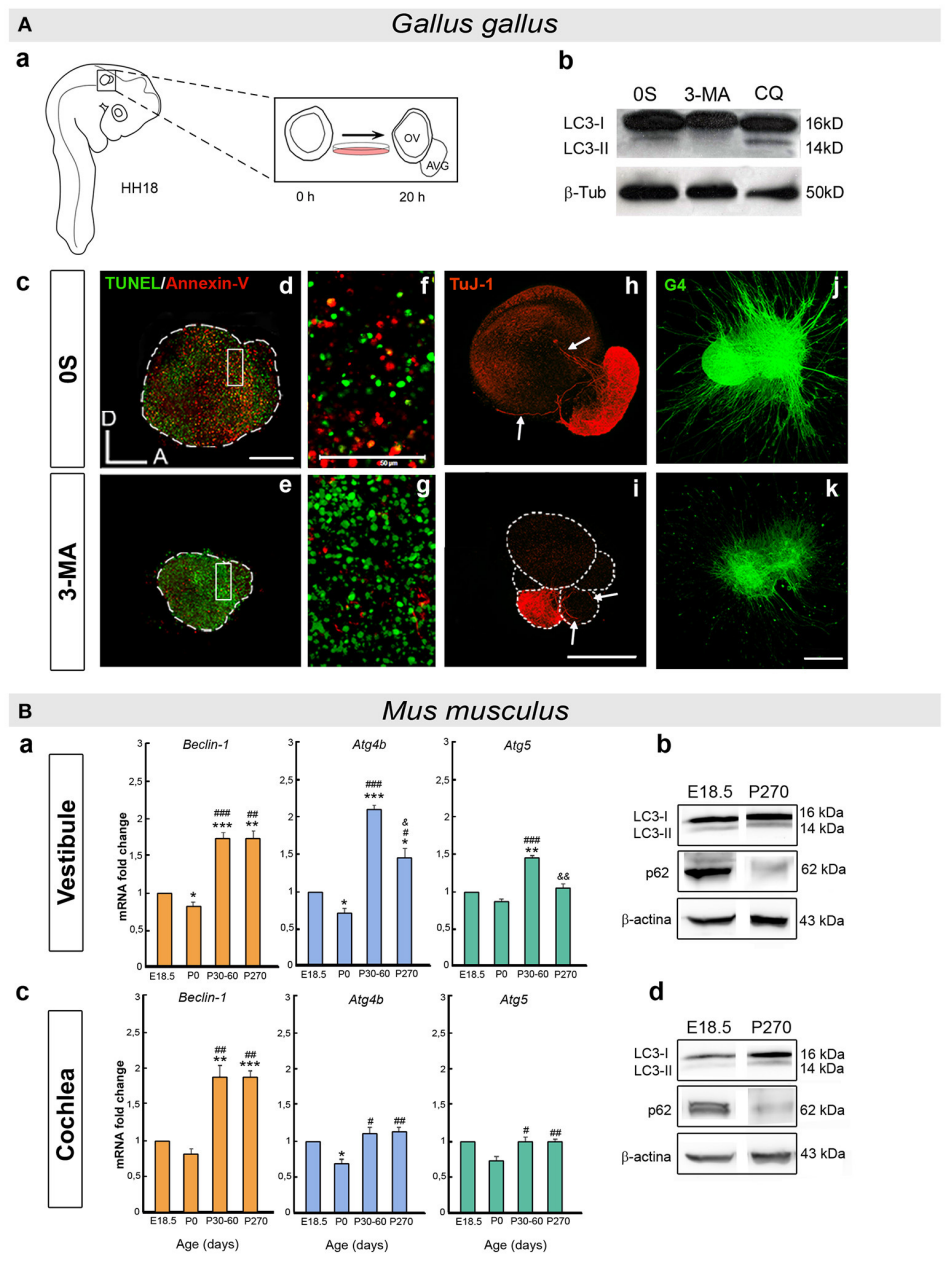
**(A)** Developmental autophagy in the chicken otocyst. **(a)** Scheme showing the *ex vivo* culture of otic vesicles from HH18 embryos. The acoustic-vestibular ganglion (AVG) develops from the cultured otic vesicle after 20 h in serum-free culture medium (0S). **(b)** Autophagic flux is typically measured in Western blots to determine the LC3 ratio in the presence or absence of chemical inhibitors of autophagy (3-MA and CQ). **(c)** Otic vesicles incubated with an inhibitor of autophagy accumulate apoptotic cells, as evident by reduced staining for An-V in red and by increased TUNEL green spots **(d–g)**. Aberrant AVG development is also seen **(h,i)**, with fewer neuroblasts (TuJ-1, red), and **(j,k)** altered neurite outgrowth and pathfinding (G4, green). **(f,g)** Higher magnification of the boxed regions in **(d)** and **(e)**, respectively. annexin-V, An-V; 3-methyladenin, 3-MA; chloroquine, CQ. Scale bars: **(d–i)**, 150 μm; **(f)**, **(g)**, 50 μm; **(j)**, **(k)** 300 μm (adapted from Aburto et al., 2012c). **(B)** Autophagy in the postnatal and adult mouse inner ear. **(a,c)** Histograms showing the changes in Beclin-1, Atg4b, and Atg5 expression with age in the mouse vestibule **(a)** and cochlea **(c)**, as determined by RT-qPCR. **(b,d)** Autophagic flux increases with age in the mouse inner ear. The LC3-II/LC3-I and SQSTM1/p62 (p62/β-actin) ratios were determined in Western blots of the vestibule **(b)** and cochlea **(d)** at E18.5 and P270. Significance: ^*^P < 0.05, ^**^P < 0.01, and ^***^P < 0.001 vs. E18.5; ^#^*P* < 0.05, ^##^*P* < 0.01, and ^###^*P* < 0.001 vs. P0; and ^&^ *P* < 0.05 and ^&&^ *P* < 0.01 vs. P30–60. E, embryonic day and P, postnatal day (adapted from de Iriarte Rodríguez et al., 2015).

Beclin-1 and Atg5 transcripts are expressed throughout the developmental stages in the chick when otic vesicles can be explanted and studied in organotypic cultures. Indeed, the Beclin-1 and LC3B proteins are present in the otic epithelium and the AVG (Aburto et al., 2012c; summarized in Figure 2A). Chemical and genetic inhibitors of autophagy demonstrate the importance of the autophagic flux for the development and cellular dynamics of the otocyst (Aburto et al., 2012c; Figure 2A). Inhibiting autophagy shows that it is required for the clearance of apoptotic cells and for cell cycle progression. Developmental apoptosis is an essential process during inner ear development (Fekete et al., 1997; Sanz et al., 1999a; Frago et al., 2003; León et al., 2004; Magariños et al., 2012), and both this cell death and the elimination of apoptotic cells require energy (Qu et al., 2007; Mellén et al., 2008). During development, autophagy provides ATP by degrading intracellular components and it thereby facilitates apoptosis. Impaired autophagy causes an accumulation of apoptotic cells that cannot be eliminated from the otic vesicle, a failing that can be reverted by adding ATP. The region where otic neural progenitors originate is the neurogenic zone, where the extracellular matrix is degraded to detach cells and the migrating detached cells accumulate autophagic vacuoles. Conversely, the inhibition of autophagy results in aberrant AVG phenotypes (Aburto et al., 2012c; Figure 2A). Therefore, autophagy is required for the migration of the epithelial neuroblasts from the neurogenic zone to form the AVG. In summary, the early development of the inner ear is one example of many where developmental autophagy plays a supporting role to apoptosis and migration (Di Bartolomeo et al., 2010; Wada et al., 2014; Lorda-Diez et al., 2015; Boya et al., 2016).

## Autophagy in the mouse inner ear

Autophagy is required for the development of the vestibular system in the mouse. *Atg4* deficient mice have impaired balance, with different phenotypic penetrance from severe to mild vestibular alterations. The biogenesis of the otoconia is defective in both *Atg4b*^−/−^ and *Atg5*^−/−^ mice (Mariño et al., 2010), and otoconial impairment occurs in mice exposed to streptomycin ototoxicity (Takumida et al., 1997), which can inhibit autophagy (Levano and Bodmer, 2015) and increase cell damage in the inner ear due to oxidative stress (Guthrie, 2008). In fact, autophagy reduces the reactive oxygen species (ROS) in mice subjected to noise-induced hearing loss (Yuan et al., 2015). This crucial role of autophagy in eliminating ROS could explain the similarities between autophagy gene mutants and streptomycin-treated animals. However, increased ROS are not the only consequence of inhibiting autophagy during vestibular development, as otoconial biogenesis requires the secretion and assembly of specific proteins that are also affected by inhibiting autophagy (Mariño et al., 2010).

Autophagy plays a key role in newborn mice, and *Atg5, Atg7, Atg9*, and *Atg16* null mice die soon after birth (Mizushima and Levine, 2010). The transcriptome of the E18.5 mouse cochlea shows that a wide variety of *Atg* genes are expressed, underlining the relevance of autophagy at perinatal stages (de Iriarte Rodríguez et al., 2015). Furthermore, several key genes of the autophagic molecular machinery (*Becn1, Atg4b, Atg5*, and *Atg9*) are expressed in the mouse vestibule and cochlea throughout development and adulthood (de Iriarte Rodríguez et al., 2015; summarized in Figure 2B). The expression of these genes is significantly enhanced from the perinatal stages (E18.5 and P0) to adulthood (P30) as the inner ear acquires its complete functionality (Rueda et al., 1996). A temporal analysis of autophagic proteolysis in the cochlea and vestibule confirms the induction of autophagy in adults rather than E18.5 embryos. Moreover, there is significantly less SQSTM1/p62 at P270 than at E.18.5, whilst the relative LC3-II levels increase in the cochlea and vestibule (de Iriarte Rodríguez et al., 2015; Figure 2B). Indeed, autophagosomes are clearly visible in adult neurons of the spiral ganglion but not at earlier stages. LC3B forms granular structures in the neuronal soma at P30 and onwards, yet not at E18.5 (de Iriarte Rodríguez et al., 2015). Autophagy is essential in neurons because they do not dilute their damaged molecules or organelles by proliferation. Thus, autophagy is required for detoxification and to manage damage (Son et al., 2012; Damme et al., 2015; He et al., 2016). Accordingly, the postnatal onset of hearing and the concomitant increase in neuronal activity is correlated with the induction of autophagy in the cochlea.

## The influence of autophagy on inner ear homeostasis and aging

Autophagy plays an additional role in inner ear homeostasis once development is concluded. Otic injury caused by a combination of aminoglycoside and loop diuretics augments aspects of autophagy (Taylor et al., 2008). Moreover, autophagy is activated by rapamycin alleviated ototoxicity in cisplatin-treated rats (Fang and Xiao, 2014) and in mice exposed to an auditory insult (Yuan et al., 2015). Thus, autophagy helps maintain adult hearing in response to stress. Proteostasis is impaired during aging (López-Otín et al., 2013) and the stabilization of proteic events that is mostly provided by molecular chaperones also declines with age (Rodriguez et al., 2016). In addition, protein degradation systems control the levels of misfolded or aggregated proteins, the accumulation of which drives age-related neurodegenerative diseases like Parkinson's or Alzheimer's disease (Balchin et al., 2016). Thus, it is not surprising that the senescence-accelerated mouse prone 8 (SAMP8) mutant mice exhibit age-related hearing loss and autophagy stress (Menardo et al., 2012).

Our studies of 9 month-old *Igf1*^−/−^ mice show they suffer defects in the proteostasis associated with aging. These *Igf1*^−/−^ mice suffer a loss of hearing and a reduced lifespan, among other traits (Varela-Nieto et al., 2013). Hearing loss in *Igf1*^−/−^ deficient mice is accompanied by a general failure of the hearing receptor (Riquelme et al., 2010), although the weaker autophagy gene expression in one-year-old cochlea may also contribute to this auditory phenotype (de Iriarte Rodríguez et al., 2015). However, the vestibular defects in the *Igf1*^−/−^ mouse are milder than those found in the cochlea (Rodríguez-de la Rosa et al., 2015). *Becn1, Atg4b*, and *Atg5* are more strongly expressed in 9-month-old *Igf1*^−/−^ vestibules compared to those of wild-type mice. Thus, the induction of autophagy might provide *Igf1*^−/−^ vestibules with some protection, as it does in *Igf1*^−/−^ retinas (Arroba et al., 2016). After differentiation, hair cells do not regenerate in the mammalian cochlea, whilst vestibular hair cells do to a limited extent (Burns and Stone, 2016). The up-regulation of autophagy might be partially responsible for the different potentiality of vestibular and cochlear hair cells.

## Conclusions

During the development of the vertebrate inner ear, autophagy participates in cell remodeling and dynamics, and it contributes to the biogenesis of the vestibular otoconia. In the postnatal cochlea, the autophagy machinery is upregulated concomitant with the increase in neuronal activity at the onset of hearing. Autophagy becomes a housekeeping process in the adult inner ear, and it is a means to protect hearing during aging and in response to injury. Further work is needed to fully understand the role of autophagy in the inner ear and to explore the potential of modulating autophagy as a novel strategy to combat inner ear diseases.

## Author contributions

MM and IV designed and drafted the work and wrote the manuscript; MM, IV, SP, MA, and RdIR analyzed and interpreted the data, revised the manuscript critically and approved the version to be published; SP designed and performed the figures. All authors are accountable for all aspects of the work in ensuring that questions related to the accuracy or integrity of any part of the work are appropriately investigated and resolved.

## Conflict of interest statement

The authors declare that the research was conducted in the absence of any commercial or financial relationships that could be construed as a potential conflict of interest.
